# A Rare Case of Hemophilia: Acquired Factor VIII Deficiency

**DOI:** 10.7759/cureus.62407

**Published:** 2024-06-14

**Authors:** Soe P Winn, Fizza Mohsin, Stephen Peeke

**Affiliations:** 1 Internal Medicine, Maimonides Medical Center, Brooklyn, USA; 2 Hematology and Oncology, Maimonides Medical Center, Brooklyn, USA

**Keywords:** acquired hemophilia, hemato-oncology, hemophilia in adult, acquired hemophilia a management, acquired hemophilia a (aha)

## Abstract

Unlike hereditary hemophilia, acquired hemophilia is a rare life-threatening bleeding disorder that occurs in a person who has no personal or family history of bleeding episodes. It usually presents with new-onset subcutaneous/joint/muscle bleeding causing ecchymoses and hematomas, hematuria, GI bleeding, retroperitoneal bleeding, or rarely acute intracranial hemorrhage in elderly individuals. The diagnosis involves assessing prolonged activated partial thromboplastin time (aPTT) and conducting mixing studies after ruling out lupus anticoagulants and interfering substances such as heparins. Management consists of controlling hemostasis using recombinant activated factor VII (rFVIIa) and activated prothrombin complex concentrates (aPCC), along with eradicating autoantibodies against factor VIII from the body system using immunosuppressive therapies. Due to the risk of uncontrolled bleeding in individuals who were previously normal, delayed diagnoses and recurrences are not uncommon, potentially resulting in unfavorable outcomes.

## Introduction

Acquired hemophilia A (AHA) is a rare life-threatening bleeding disorder caused by malfunctioning factor VIII due to the abnormal production of autoantibodies against factor VIII [1,2]. Physiologically, factor VIIIa functions as a vital cofactor, forming a tenase complex with factor IXa and calcium in the intrinsic pathway. This complex activates factor X to factor Xa, which is essential for the downstream activation of the common pathway involving prothrombin, fibrinogen, and factor XII in the coagulation cascade. Additionally, factor VIII undergoes proteolytic cleavage leading to the removal of a significant portion of the B domain, resulting in the formation of a heterodimer that tightly circulates in complex with von Willebrand factor (vWF) through the heterodimerization of heavy (A1 and A2 domains) and light (A3, C1, and C2 domains) chains. In the event of vascular insults, additional proteolytic processing of the complex generates activated factor VIIIa (fVIIIa), a heterotrimer (A1/A2/A3-C1-C2) that dissociates from vWF and binds to activated platelets [3,4]. In AHA, these autoantibodies specifically bind to A2, A3, or C2 domains with epitope specificity, preventing interaction with vWF, thrombin, activated factor IX, and/or factor X [4-6]. It is also known that some of these autoantibodies can possess proteolytic properties that decrease the factor VIIIa levels in the blood, resulting in the impairment of both the quantity and quality of factor VIIIa [7].

According to Gawryl and Hoyer, these autoantibodies, primarily of the IgG-type, attack the A2, A3, and C2 domains. Anti-C2 inhibitors disrupt the binding of FVIII to phospholipids and vWF, while A2 and A3 inhibitors impede the binding of FVIII to factor X (FX) and FIXa, respectively, hindering the formation of the Xase complex. They bind to FVIII in a type II pharmacokinetic fashion, resulting in initial non-linear rapid inactivation, followed by a slower phase of inactivation, leading to the incomplete inhibition of FVIII activity. Consequently, FVIII activity is not completely suppressed, displaying a partial inhibition pattern due to these autoantibodies. This differs from type I pharmacokinetics, where FVIII activity is completely inactivated, as observed in hemophilia A patients. They acquire alloantibodies from multiple blood transfusions, and these alloantibodies can totally abolish the factor VIII activity [1,5,8]. 

In contrast to hemophilia A which can result in the development of alloantibodies in approximately 5-20% of severe cases with repeated blood transfusion, acquired hemophilia is estimated to occur in one in a million population per year. However, the exact incidence is not known due to the rarity of the disease, and most data come from retrospective case reports or case series [5,9]. It typically affects elderly individuals in an equal male-to-female ratio, unlike the X-link recessive male-predominant fashion seen in congenital hemophilia A [9,10].

While approximately 50% of the AHA cases do not have any underlying disease process, it can be associated with autoimmune disorders (systemic lupus erythematosus, rheumatoid arthritis, Sjögren's syndrome, etc.), malignancy (lymphoproliferative malignancies), pregnancy, and postpartum period. Some immune checkpoint inhibitors (pembrolizumab, etc.) are reported to have AHA after the initiation of the treatment, but underlying mechanisms are not fully understood [1,8,11]. It is postulated that AHA is closely linked with certain polymorphisms in CTLA4, F8 gene, or DRB1*15 HLA class II [7,12].

AHA typically presents with new-onset symptoms of subcutaneous, joint, or muscle bleeding, hematuria, GI bleeding, retroperitoneal bleeding, or, rarely, sudden acute intracranial hemorrhage [10,13]. The diagnosis entails evaluating prolonged activated partial thromboplastin time (aPTT) and performing mixing studies following the exclusion of lupus anticoagulants and interfering substances like heparins. Differentials comprise prolonged aPTT attributable to vitamin K deficiency, anticoagulants, antiphospholipid syndrome, disseminated intravascular coagulation, acquired vWF deficiency, etc. Due to the possible uncontrolled bleeding in individuals without a previous history of bleeding disorder, delayed diagnosis and management are common, which can lead to serious morbidity and mortality in these patients.

According to the European Acquired Hemophilia Registry (EACH2), the average time to diagnosis is within one week. However, in a study conducted by Pardos-Gea et al., the median time to first diagnosis is 19 days, primarily due to the use of anticoagulants [14]. After the discontinuation of immunosuppressive therapy, recurrences are not uncommon, with approximately 25% of patients experiencing a relapse, having a median time to relapse of 14.7 weeks. Mortality rates range from 6.7% to 38%, attributed to fatal bleeding from the disease itself and infections due to immunosuppressants [15]. Therefore, we would like to present a case of AHA who presented with new-onset symptoms of easy bruising, right knee pain, and swelling.

## Case presentation

An 83-year-old male with a medical history of hypertension, diabetes, benign prostatic hyperplasia, chronic kidney disease, osteoarthritis, and chronic venous insufficiency presented with his right knee feeling "firm" for a week. The patient also complained of easy bruising recently even though there was no history of falls or trauma. He denied any recent illness/infection, hemoptysis, hematemesis, abdominal pain, and frank blood in urine or stool.

Otherwise, review of systems was negative. There was no personal or family history of bleeding disorders. According to the patient, this was a relatively new symptom despite taking aspirin for many years. He did not take any other antiplatelets or anticoagulation. His other medications are amlodipine, losartan, atorvastatin, metformin, and tamsulosin. At baseline, he uses a cane to walk because of osteoarthritis of the knee; however, he can walk 3-4 blocks without experiencing pain or shortness of breath. On examination, the patient was hemodynamically stable. Physical examination revealed significant for multiple 5-6 ecchymoses of 1-2 cm in size on both upper extremities and a swollen right knee with a decreased range of motion. Laboratory tests were significant for normocytic anemia with a hemoglobin (Hgb) of 6.7 (baseline was 11.1). His international normalized ratio (INR) was 1.3, and his aPTT was more than 100 seconds with a normal platelet count of 450k. The hemolytic workup such as haptoglobin, lactate dehydrogenase (LDH), and peripheral blood smear was unremarkable. Serum protein electrophoresis (SPEP)/serum immunofixation and hepatitis labs were negative as were signs of autoimmune vasculitis. Imaging of the right knee was consistent with joint effusion. Whole-body CT scans were negative for any lymphoproliferative disorder or underlying malignancy.

**Figure 1:**
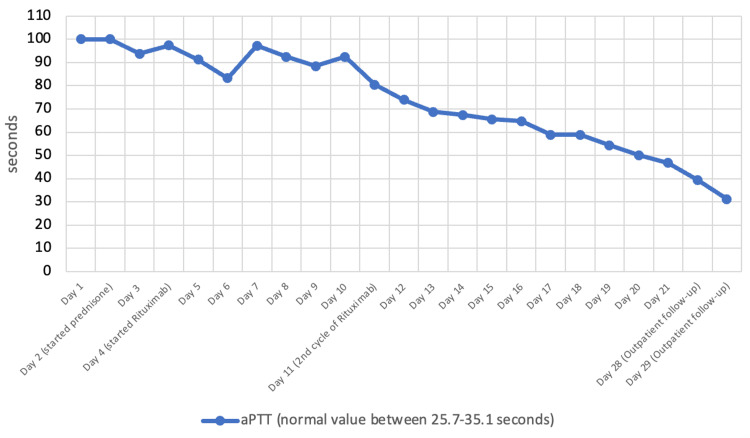
aPTT response to prednisone and rituximab therapy aPTT: activated partial thromboplastin time

Aspirin was immediately discontinued. Supportive transfusions and vitamin K were administered initially due to a very high aPTT of >100 seconds. After administering four units of fresh frozen plasma (FFP), aPTT was consistently >100 seconds and did not correct with mixing studies after ruling out lupus anticoagulants, suggesting acquired hemophilia. The blood bank was contacted to start the patient on the bypassing agent rFVII 90 mcg/kg every four hours along with steroids. Factor VIII levels were less than 1, with a high titer of factor VIII inhibitor, 438.5 Bethesda unit (BU/ml), reported (factor VIII inhibitor level in a normal individual is normally less than 0.5 BU/ml). He was diagnosed with idiopathic acquired hemophilia and started on rituximab 375 mg/m^2^ IV once weekly for four weeks according to the GTH registry study, alongside supportive rFVII therapy. rFVII was later replaced with IV tranexamic acid as aPTT slowly improved after treatment (Figure 1). He was also prescribed Bactrim for *Pneumocystis jirovecii* pneumonia (PJP) prophylaxis due to prolonged steroid use while monitoring his potassium and kidney function at outpatient follow-ups. The patient was discharged on a steroid taper regimen with outpatient follow-up at the hematology clinic, and he continued to do well with a normal aPTT.

## Discussion

Diagnosis of AHA is a complex, multi-step process that necessitates clinical suspicion and a thorough evaluation of patient history and physical examination. In the literature reviews, most AHA patients present with acute onset of bleeding or ecchymoses [1,9,10]. In our case, the patient is an elderly man with no previous history of bleeding or clotting disorders, who presented with a new onset of easy bruising and ecchymoses. His medical history was negative for an autoimmune disorder, solid cancer or lymphoproliferative disorder, chronic bleeding/clotting disorder, and a family history of hemophilia, and he was not taking any new blood thinners or medications.

If the laboratory evaluation revealed normal platelet counts and prolonged aPTT levels on repeated tests, one should obtain a mixing study using a 50:50 mix of patient plasma with normal plasma including a two-hour incubation at 37°C, factor VIII activity, and Nijmegen-modified Bethesda assay for antibody titer level. In the mixing study, if an inhibitor exists, the aPTT remains prolonged. Conversely, in the absence of an inhibitor, the factor amount in the control plasma adequately corrects the aPTT to the normal range. Other coagulation factor levels and vWF levels, antiphospholipid antibodies, autoimmune tests such as rheumatoid factor, ANA panels, and viral panels such as Epstein-Barr virus (EBV), cytomegalovirus (CMV), and hepatitis profiles should also be tested to rule out the other coagulation factor deficiencies or possible underlying causes.

If either the mixing study or factor VIII activity is suggestive of AHA, treatment for bleeding control can be initiated while awaiting confirmatory test results.

Management of the AHA involves the management of hemostasis with bypassing agents and the eradication of autoantibodies with immunosuppressive therapy. Due to the rarity of the disease, the management of such cases relies on the 2020 international consensus recommendation rather than a specific management guideline [16].

If active bleeding is suspected, the recommendations are to start on recombinant activated factor VII (rFVIIa), prothrombin complex concentrate (PCC), or recombinant porcine factor VIII instead of human FVIII concentrates or desmopressin (DDAVP). The decision should not depend on the antibody titer level or FVIII activity because of the type II pharmacokinetic as we explained above. New medications like emicizumab, which is a bispecific IgG4 antibody that binds to factor IXa and factor X, mimic the function of normal FVIII. It is registered for bleeding prophylaxis in congenital hemophilia A but lacks data in AHA patients.

For the eradication of autoantibodies, immunosuppressive therapy is recommended by 2020 international recommendation. Steroids (prednisolone) should be started if the Bethesda unit is less than 20 BU/ml with FVIII activity ≥1 IU/dL, with rituximab or cyclophosphamide added if the patient is not responding well to steroids. If Bethesda units exceed 20 BU/ml with FVIII activity ≥1 IU/dL, the GTH registry study recommends combining steroids with cyclophosphamide or rituximab. The study found that achieving partial remission within 21 days of steroid therapy was improbable, with a negative predictive value of 84%, for patients presenting with FVIII levels below 1% or an inhibitor titer exceeding 20 BU [17].

CyDRi (cyclophosphamide, dexamethasone, and rituximab) combination therapy was assessed in a retrospective cohort study involving 32 patients across two institutions. The outcomes revealed a satisfactory result, with a durable complete response rate of 96.9%. However, treatment-related complications, such as infections stemming from cytopenia, were observed [18]. Over the last 20 years, the anti-CD20 monoclonal antibody rituximab has been effective in the management of certain autoimmune diseases and cancers. There have been retrospective studies performed on treating AHA patients who have high Bethesda unit (>100BU/ml) with rituximab, and in these patients, rituximab therapy has been an effective agent in achieving a similar response (complete response or partial remission) with a better safety profile than other immunosuppressive agents [19-21]. Intravenous immunoglobulin (IVIG) therapy and single-agent mycophenolate mofetil or cyclophosphamide were also investigated, but these medications need comparative study with currently recommended regimens [22,23]. 

## Conclusions

AHA is a rare coagulation disorder, affecting approximately one in a million people, and may entail a significant mortality rate due to its prevalence among elderly patients with multiple comorbidities, often including the use of multiple medications, including blood thinners. Consequently, the manifestation of AHA can mimic other bleeding or clotting disorders or medication side effects, potentially leading to delayed diagnosis and unfavorable patient outcomes. Therefore, when the aPTT level is abnormal, it is crucial to determine the underlying cause, given the broad and potentially life-threatening differential diagnosis, which includes conditions such as vitamin K deficiency, anticoagulants, antiphospholipid syndrome, disseminated intravascular coagulation, and acquired vWF deficiency. The diagnostic algorithm begins with evaluating prolonged aPTT and conducting mixing studies while ruling out lupus anticoagulants and inquiring about medications or substances that can prolong aPTT levels. Maintaining a high level of clinical suspicion, conducting timely investigations, involving a multidisciplinary team, and promptly initiating treatment are crucial for managing AHA patients, particularly in complex cases with multiple comorbidities. Further research and evidence are crucial to improving the management of AHA, particularly through the exploration of combination therapies and the development of novel approaches.
